# Supplementary material for: WordCommentsAnalyzer: A windows software tool for qualitative research
Source: F1000Res. 2018 Sep 4;7:536. Originally published 2018 May 3. [Version 2] doi: 10.12688/f1000research.14819.2 (PMC6137411; doi:10.12688/f1000research.14819.2)
Supplement: Supplementary file 3 [file f1000research-7-17614-s0002.tgz › b2a79496-4ada-47c4-9682-a99ce867836d.zip!/Eight Iranian Health Journals/Journal of Research in Rehabilitation Sciences _20.docx]

© Journal of Research in Rehabilitation Sciences . This work is licensed under CC BY-NC 4.0 (https://creativecommons.org/licenses/by-nc/4.0/).

Volume & Issue: دوره 10، شماره 5: 1393

Title: بررسی تأثیر کنترل بینایی بر ثبات وضعی ایستایی بیماران مبتلا به پارکینسون خفیف با دو رویکرد خطی و غیر¬خطی

http://jrrs.mui.ac.ir/index.php/jrrs/article/view/1650

چکیده مقدمه: بیماری پارکینسون اختلالی مزمن در هسته­های قاعده­ای مغز است و بصورت پیشرونده­ عملکرد حرکتی و غیرحرکتی مبتلایان را تحت تأثیر قرار می­دهد. ترمور حین استراحت، برادی کینزیا، ریجیدیتی و بی ثباتی وضعی علائم اصلی بیماری پارکینسون محسوب می­شوند. مطالعات مختلفی به بررسی ثبات وضعی بیماران مبتلا به پارکینسون خفیف در حالت ایستاده بر دو پا با استفاده از سکوی اندازه­گیری نیرو پرداخته اند اما تا آنجا که نویسندگان مقاله مطلع اند اجماع نظری بر کیفیت ثبات وضعی ایستایی این بیماران در وجود ندارد لذا هدف از مطالعه حاضر ارزیابی ثبات وضعی ایستایی بیماران مبتلا به پارکینسون خفیف با دو رویکرد خطی و غیر­خطی آنالیز پارامترهای مرکز فشار می­باشد. همچنین بررسی تأثیر کنترل حس بینایی بر تعادل ایستایی این بیماران از اهداف دیگر مطالعه است. مواد و روش­ها: هفده نفر با شدت خفیف (Hoehn/Yahr scale: 1) بیماری پارکینسون و 17 فرد سالم همسان مورد مطالعه قرار گرفتند. تست ها بصورت 60 ثانیه ایستادن بر سکوی اندازه­گیری نیرو و در حالات چشم باز و بسته انجام شد. دامنه، طول مسیر، سرعت و ریشه میانگین مربعات مرکز فشار در دو راستای قدامی/خلفی و داخلی/خارجی، پارامترهای خطی و آنتروپی تقریبی متغیر غیر خطی مورد محاسبه مرکز فشار بود. جهت آنالیز نهایی اطلاعات از آزمون Mixed ANOVA (2×2) استفاده گردید.  یافته­ها: مقدار میانگین پارامترهای طول مسیر و سرعت مرکز فشار در راستای قدامی/خلفی، داخلی/خارجی و کلی به طور معناداری در گروه بیمار بیشتر از گروه سالم بود و با بستن چشمان در هر دو گروه افزایش قابل ملاحظه یافت. مقدار میانگین آنتروپی تقریبی قدامی/خلفی و داخلی/خارجی مرکز فشار در گروه بیمار بیشتر از گروه سالم بود اما تفاوت معناداری نداشت. البته با حذف اطلاعات حس بینایی در هر دو گروه بیمار و کنترل افزایش قابل ملاحظه یافت. نتیجه­گیری: بیماران در مراحل ابتدایی پارکینسون دارای یک بی ثباتی وضعی ایستایی ساب کلینیکال می­باشند. البته جهت کنترل تعادل ایستایی خود به حس بینایی وابسته نیستند.

Title: تأثير هشت هفته ماساژدرماني بر كيفيت زندگي بيماران زن مبتلا به مولتيپل اسكلروزيس

http://jrrs.mui.ac.ir/index.php/jrrs/article/view/1811

مقدمه: بيماري MS يكي از شايع ترين بيماري هاي مزمن سيستم اعصاب مركزي است. اين مطالعه با هدف تعيين تأثير 8 هفته ماساژ درماني بر کيفيت زندگي زنان مبتلا به MS انجام گرفت. مواد و روش ها: در اين پژوهش نيمه تجربي، 20 نفر از زنان مبتلا به MS مراجعه کننده به انجمن MS اصفهان به روش در دسترس هدفمند انتخاب شدند. شرط ورود به مطالعه ابتلا به MS عود ـ­ فروکش کننده با EDSS كمتر از 5/3 بود. بدون آنکه تغييري در برنامه درماني بيماران داده شود، آنها به صورت تصادفي جفت  شده در 2 گروه شاهد (10نفر) و تجربی (10نفر) قرار گرفتند. پس از يك جلسه توجيهي از كليه آزمودني ها رضايت نامه كتبي اخذ گرديد. پروتکل ماساژ درماني منطبق بر متد روسي بود و به مدت 2ماه (2 جلسه 30 دقيقه­اي در هفته) در گروه تجربی انجام گرفت. كيفيت زندگي بيماران قبل و بعد از دوره تمريني توسط پرسشنامه QOL_54 اندازه گيري شد. داده ها با استفاده از تحليل واريانس براي داده هاي تکراري مورد تجزيه و تحليل قرار گرفتند. سطح معنادارای برای کلیه تحلیل ها 05/0 در نظر گرفته شد. يافته ها: تمامي عوامل اندازه­گيري شده در تحقيق شامل كيفيت زندگي کلي، سلامت جسماني، و سلامت رواني در بيماران داراي تعامل معنادار بود(004/0p≤ و 98/12≤(18و1)F). بحث: بيماران هر دو گروه تحقيق تحت تاثير مراقبت­هاي پزشکي بودند، اما فقط گروه ماساژ در ابعاد مختلف کيفيت زندگي پيشرفت بيشتري را نشان داد. اين يافته­ ها مويد استفاده از ماساژ درماني به عنوان يک مداخله غير تهاجمي، کم هزينه و مکمل براي بيماران زن مبتلا به MS مي­باشد.

Title: تأثیر کفی بافت دار بر تعادل در افراد با بی ثباتی عملکردی مچ پا

http://jrrs.mui.ac.ir/index.php/jrrs/article/view/1996

مقدمه: اختلالات تعادل یکی از مشکلات مهم در افراد با بی ثباتی عملکردی مچ می باشد که ارتباط آن با آسیب گیرنده های مکانیکی مفصل گزارش شده است. کفی بافت دار با تاثیر بر روی پوست پلانتار پا ها ممکن است تعادل را بهبود بخشد و ریسک آسیب در افراد با بی ثباتی عملکردی را کاهش دهد. بنابراین هدف این مطالعه بررسی تاثیر کفی بافت دار بر تعادل ایستادن در افراد با بی ثباتی عملکردی بود. مواد و روش ها: تعادل ایستادن بر روی دو پا در 20 ورزشکار با علائم بی ثباتی عملکردی با استفاده از صفحه نیرو طی دو مداخله: 1) کفی ساده 2) کفی بافت دار از جنس اتیل ونیل استات تست شد. متغیرهای تعادل ایستادن شامل: متوسط مسافت طی شده مرکز فشار، متوسط سرعت مرکز فشار در صفحات قدامی- خلفی و داخلی خارجی، مساحت ناحیه دایره ای محاط بر محدوده ی حرکت مرکز فشار( با اطمینان 95درصد) و مساحت ناحیه چرخش مرکز فشار در واحد زمان بود. تحلیل آماری داده ها با استفاده از تست آماری nonparametric Wilcoxon Signed Rank انجام گرفت. یافته ها: همه متغیرهای تعادل ایستادن حین پوشیدن کفی بافت دار در مقایسه با کفی ساده کاهش یافتند، اما فقط از نظر آماری کاهش معناداری در مساحت ناحیه دایره ای محاط بر محدوده ی حرکت مرکز فشار( با اطمینان 95درصد) بدست آمد (<0.05). نتیجه گیری: استفاده از بافت روی کفی ساده در افراد با بی ثباتی عملکردی ممکن است تا حدودی روی تعادل ایستادن تأثیر گذار باشد.

Title: : اختلالات حسی در پای مبتلایان به مولتیپل اسکلروزیس و ارتباط آن با تعادل و کیفیت زندگی این افراد

http://jrrs.mui.ac.ir/index.php/jrrs/article/view/2056

مقدمه: اختلالات حسی از عوارض مهم ابتلا به مولتیپل ­اسکلروزیس محسوب می­گردد. براساس شواهد موجود در افراد سالمند و نیز افراد مبتلا به دیابت و یا بیماری­های نورولوژیک سیستم اعصاب­مرکزی (مثل سکته­مغزی)، کاهش حس ناحیه پا می­تواند اثرات منفی بر تعادل، تحرک و کیفیت­ زندگی افراد به­ جای گذارد؛ با این وجود مطالعات روی افراد مبتلا به مولتیپل­ اسکلروزیس کمتر بر کیفیت حس ناحیه پا متمرکز شده ­است. هدف از این مطالعه بررسی شیوع اختلالات­ حسی در پای افراد مبتلا به مولتیپل­اسکلروزیس و تاثیر آن بر تعادل و کیفیت­ زندگی این افراد می­باشد. مواد و روش­ها: در یک مطالعه مقطعی 60 فرد مبتلا به مولتیپل ­اسکلروزیس و یک گروه 30 نفره از افراد سالم شرکت کردند. ارزیابی حسی این افراد شامل بررسی حس ­ظریف پای افراد و حس تشخیص موقعیت­ مفاصل مچ­ پا و اولین متاتارسوفالنژیال صورت­گرفت. و از تست­های حداکثر دسترسی قدامی(FRT) و سنجش زمان بلند­شدن از روی صندلی و راه­ رفتن (TUG) برای ارزیابی تعادل افراد استفاده­شد. نهایتا برای بررسی کیفیت­زندگی افراد پرسشنامه SF-36 تکمیل­گردید. نتایج: نتایج نشان­داد که ٪3/68 از افراد مبتلا به مولتیپل­اسکلروزیس از نظر حس­ ظریف و ٪3/63 از آنها از نظر حس­ عمقی دچار درجاتی از اختلالات­ حسی می ­باشند. در مقایسه متغیرهای تعادلی نیز تفاوت­های بارزی میان افراد مبتلا به مولتیپل­اسکلروزیس و گروه کنترل مشاهده­شد. بررسی همبستگی­ها میان فاکتورهای­ حسی و تعادلی و کیفیت­زندگی حاکی از وجود روابط معنادار بود (01/0P<). بحث: مقایسه داده­های حاصل از آزمون­های حسی، تعادلی و کیفیت­ زندگی بیانگر وجود تفاوت­های بارز میان افراد مبتلا به مولتیپل­اسکلروزیس  و افراد سالم بود(01/0P<). مهمترین یافته حاصل از این کار حاکی از شیوع نسبتا بالای اختلالات­­ حسی در پای­افراد مبتلا به مولتیپل­اسکلروزیس و وجود ارتباطات میان اختلالات­ حسی با تعادل و کیفیت­ زندگی در این افراد می­باشد. بر اساس این نتایج توصیه می­گردد که در طرح برنامه­های توانبخشی جهت بهبود شرایط تعادل و ارتقای کیفیت زندگی مبتلایان به مولتیپل­اسکلروزیس، به ارزیابی حس پا و درمان مشکلات آن توجه ویژه شود.

Title: تأثیر شدت استئوآرتریت زانو بر پارامترهای زمانی- مکانی راه رفتن زنان ـ یک مطالعه مقدماتی

http://jrrs.mui.ac.ir/index.php/jrrs/article/view/2093

مقدمه: استئوآرتریت زانو یکی از شایع‌ترین بیماری‌های مفصلی وابسته به سن است. شدتهای مختلف این بیماری از طریق مکانیزم‌های جبرانی مختلف منجر به تغییر در الگوی راه رفتن می‌شود. هدف مطالعه حاضر بررسی چگونگی تأثیر شدتهای مختلف استئوآرتریت زانو بر پارامترهای زمانی ـ مکانی راه رفتن است. مواد و روش‌ها: 40 زن در سه گروه استئوآرتریت خفیف٬ متوسط و شدید و یک گروه افراد بدون استئوآرتریت بر اساس معیار K-L Scale برای این مطالعه انتخاب شدند. افراد با سرعت مورد دلخواه خود در مسیر تعیین شده آزمایشگاه راه رفتند تا 5 آزمون موفق ثبت گردید. سپس پارامترهای زمانی ـ مکانی راه رفتن شامل طول گام ، سرعت، آهنگ راه رفتن، درصد فاز استانس، زمان گام و زمان استانس مورد آنالیز قرار گرفتند. يافته‌ها: نتايج تستهاي ANOVA و آناليز Post hoc نشان داد که طول گام و طول گام اصلاح شده تنها پارامترهايي بودند که در گروه سالم تفاوت معني‌داري با گروه استئوآرتريت متوسط و شديد داشتند (004/0=P و 001/0=P براي طول گام و 014/0=P و 001/0=P براي طول گام اصلاح شده). گروه استئوآرتريت شديد در تمام متغيرها با گروه‌هاي کنترل و استئوآرتريت خفيف داراي تفاوت معني‌دار بود (05/0>P). به علاوه زمان گام در افراد داراي استئوآرتريت متوسط به‌طور معني‌داري کمتر از افراد استئوآرتريت شديد بود (038/0=P). نتيجه‌گيري: به نظر مي‌رسد متغيرهاي زماني ـ مکاني راه رفتن پارامترهاي ارزشمندي براي تفکيک شدت‌هاي مختلف استئوآرتريت زانو باشند و مي‌توانند اين بيماران را از افراد سالم تفکيک کنند. از بين اين پارامترها احتمالا طول گام يک پارامتر مناسب براي تشخيص افراد بدون استئوآرتريت زانو از افرادي است که از اين بيماري رنج مي‌برند.

Title: اثر روش¬های مختلف کششی در طول گرم کردن بر تعادل ایستا و پویای دانشجویان تربیت بدنی دختر

http://jrrs.mui.ac.ir/index.php/jrrs/article/view/1305

مقدمه: هدف از این تحقیق، بررسی اثرات روش­های کششی ایستا، پویا و بدون کشش در طول گرم کردن بر تعادل ایستا و پویای دانشجویان تربیت بدنی دختر بود. مواد و روش­ها:  18 دانشجوی تربیت بدنی دختر (قد: 5.94 ± 159.68 سانتیمتر؛ جرم: 5.93 ± 60.21 کیلوگرم؛ سن: 0.63 ± 22.00 سال) برای عملکرد تعادل ایستا با آزمون لک­لک و همچنین برای تعادل پویا بوسیلۀ آزمون ستاره بعد از روش­های مختلف کششی که شامل کشش ایستا، کشش پویا و بدون کشش بود، سنجیده شدند. یافته­ها: افزایش معناداری در تعادل ایستا پس از کشش پویا (3.14 ± 6.34 ثانیه) نسبت به کشش ایستا (2.47 ± 4.40 ثانیه، 0.03=P) وجود داشت. بعلاوه، افزایش معناداری در نمرات دستیابی تعادل پویا پس از کشش پویا (3.76 ± 72.03 درصد طول پایین تنه) نسبت به کشش ایستا (5.35 ± 69.58 درصد طول پایین تنه ، 0.01=P) وجود داشت. نتیجه گیری: محققان نتیجه گرفتند که برای حفظ تعادل، ورزشکاران بهتر است از روش کششی پویا استفاده کنند و بدن خود را هر چه بیشتر به این نوع روش کششی سازگارتر کنند چون این نوع روش کششی موجب افزایش عملکرد تعادل می­شود.

Title: تأثیر پروتکل تمرینی، بر تعادل پویا در هنگام راه رفتن بر روی چوب موازنه در دانش آموزان 11 تا 13 سال

http://jrrs.mui.ac.ir/index.php/jrrs/article/view/1984

چکیده مقدمه: هدف از این تحقیق تأثیر پروتکل تمرینی، بر تعادل پویا در هنگام راه رفتن بر روی چوب موازنه در دانش آموزان 11 تا 13 سال بود. مواد و روش­ ها: 30 دانش آموز غیر ورزشکار پسر به طور تصادفی به دو گروه تجربی و کنترل تقسیم شدند. گروه تجربی به مدت هشت هفته در یک پروتکل تمرینی شرکت کردند. قبل و بعد از پروتکل تمرینی عملکرد تعادلی آن­ها با استفاده از راه رفتن روی چوب موازنه اندازه­گیری و مقایسه شد. تغییر موقعیت شانه، لگن و مچ پا توسط دستگاه تجزیه و تحلیل حرکتی اندازه­گیری شد. برای تجزیه تحلیل آماری از روش آماری تی وابسته و مستقل در سطح معناداری 05/0 استفاده شد. یافته­ ها: نتایج کاهش معناداری در تغییر موقعیت شانه در گروه تجربی قبل و بعد از پروتکل تمرینی و کاهش معناداری در تغییر موقعیت مچ پا در گروه تجربی و کنترل نشان داد ولی تفاوت معناداری در تغییر موقعیت شانه در گروه کنترل و تجربی، تغییر موقعیت لگن در گروه کنترل و تجربی بعد از پروتکل تمرین و تغییر موقعیت لگن در گروه تجربی بعد از پروتکل تمرین و تغییر موقعیت مچ پا در گروه تجربی قبل و بعد از پروتکل تمرین مشاهده نشد. نتیجه­ گیری: به طور کلی نتایج نشان می­دهند که تمرینات تعادلی تخصصی بالانس روی دست بیشتر سبب پیشرفت تعادل پویا در اندام فوقانی می­شوند.

Title: ارزیابی متغیر های فضایی زمانی و نیروی عکس‌العمل زمین هنگام راه رفتن در بیماران پارکینسون و افراد سالم

http://jrrs.mui.ac.ir/index.php/jrrs/article/view/1968

مقدمه: بیماری پارکینسون با ناهنجاری­های حرکتی از جمله تغییر الگوی راه رفتن همراه است. هنوز اطلاعات موجود در زمینه ویژگی­های کینتیکی راه رفتن در این بیماران ناکافی است. هدف این تحقیق ارزیابی ویژگی‌های فضایی-زمانی و نیروی عکس‌العمل زمین هنگام راه رفتن در بیماران پارکینسون و مقایسه آن با افراد سالم بود. مواد و روش‌ها: تعداد 14 مرد مبتلا به بیماری پارکینسون با دامنه سنی 50 تا 75 ساله و 16 مرد سالم با دامنه سنی مشابه مورد مطالعه قرار گرفتند. با استفاده از یک سیستم تحلیل حرکتیVicon    (100 Hz) با چهار دوربین سری T و دو دستگاه صفحه نیروی Kistler (1000 Hz) متغیر های فضایی زمانی و نیروی عکس‌العمل زمین در مرحله استقرار راه رفتن اندازه­گیری شدند. بعد از حذف نویز و پردازش داده­ها برای تحلیل آماری از آزمون Independent t-test در محیط نرم افزار SPSS-19 و 05/0 <α استفاده شد. نتایج: سرعت راه رفتن، طول گام، اوج دومین نیروی عمودی، و نیروی عکس­العمل عقبی-جلویی (جلو برنده) در بیماران به طور معنی داری کمتر از گروه کنترل بود(05/0p<). مدت زمان حمایت دوگانه در بیماران بیشتر بود(05/0p<). نیروهای عکس­العمل زمین در پای چپ و راست در هر دو گروه قرینه بود(05/0p>). بحث: کاهش سرعت راه رفتن و افزایش فاز حمایت دوگانه در افراد پارکینسون ناشی از نوعی انطباق سیستم عصبی-عضلانی برای جبران ضعف تعادل باشد. قله دوم نیروی عکس‌العمل عمودی (Fz3) با نیروهای عکس­العمل عقبی-جلویی (پیش برنده) ارتباط مستقیم دارد و کاهش این نیروها در بیماران پارکینسون ممکن است به دلیل کاهش سرعت راه رفتن ‌باشد. البته راه رفتن آهسته در دراز مدت ممکن است خود موجب ضعف عضلانی گردد.

Title: بررسی عوامل مؤثر بر مصرف انرژی در آمپوته های زیر زانو در طی راه رفتن؛ مقاله¬ی مروری

http://jrrs.mui.ac.ir/index.php/jrrs/article/view/1867

مقدمه: اندازه گیری مصرف انرژی، یکی از روش های مؤثر جهت ارزیابی کارآیی پروتز در افراد قطع عضو اندام تحتانی می باشد. هر قطعه پروتزی که بتواند مصرف انرژی فرد را کاهش داده و در عین حال، سبب افزایش راحتی بیمار شود، بهترین انتخاب برای این گروه می باشد. بنابراین، هدف از این مطالعه، ارزیابی میزان مصرف انرژی در افراد قطع عضو زیر زانو و عوامل مؤثر بر آن می باشد. مواد و روش ها: این مطالعه به روش مروري و با استفاده از واژه­هاي کلیدي below knee amputees ، transtibial amputee، Energy expenditure ، Oxygen consumption،  walkingانجام شد. جست و جوی مقالات در پایگاه­های اطلاعاتی pubmed، Cochrane library، Google scholar و ISI web of science از سال های 1970 تا 2014 میلادی صورت گرفته است. یافته ها: تعداد 76 مقاله بدست آمد که پس از بررسی عناوین و چکیده­ مقالات، تعدادي از آنها به دلیل عدم ارتباط با اهداف مطالعه حذف گردید. سپس 20 مقاله­ واجد شرایط انتخاب شد و مورد تجزیه و تحلیل قرار گرفت و در پایان، نتیجه گیري کلی به عمل آمد. امتياز گزارش دهي، روايي خارجي، روايي داخلي (بایاس) و روايي داخلي مخدوش کننده به ترتيب بين 9-4، 3-2، 5-4 و 5-3 متغیر بود. نتیجه گیری: این مطالعه مروری نشان می دهد که مصرف انرژی آمپوته های زیر زانو در طی حرکت از افراد سالم بیشتر است. این موضوع تحت تأثیر عوامل مختلفی شامل سرعت راه رفتن، سطح قطع عضو و الایمنت پروتز قرار دارد. بر خلاف تصور رایج، شواهد موجود نشان می دهد که وزن پروتز، نوع پنجه و طول استمپ، تأثیر معنی داری بر مصرف انرژی ندارد. در مطالعات انجام شده به تأثیر نوع تعلیق پروتز و مناسب بودن فیتینگ آن، بر مصرف انرژی پرداخته نشده است. در مورد مصرف انرژی آمپوته ها، انجام تحقیقاتی با جامعه آماری بزرگتر که دربردارنده انواع مختلف قطع عضو زیر زانو باشد، لازم به نظر می رسد.

Title: روش های ارزیابی داستان گویی در کودکان: یک مرور نظام مند

http://jrrs.mui.ac.ir/index.php/jrrs/article/view/2090

مقدمه: توانایی های داستان گویی در کودکان را می توان پیشگوی خوبی از مهارتهای خواندن و عملکرد تحصیلی دانست. ارزیابی مهارت داستان گویی در دو بخش ارزیابی ساختارهای بزرگ (macro)  و کوچک (micro) انجام می شود. در ارزیابی ساختارهای بزرگ، مهارت های توصیف عمل، نتیجه و تحلیل  و در ارزیابی ساختارهای کوچک، پیچیدگی نحوی و دستوری در داستان کودک بررسی می شود. این مطالعه با مروری هدفمند بر آخرين مقالات و كتاب هاي منتشر شده در زمینه ی داستان گویی به  مقایسه ی محتوایی روش های مختلف ارزیابی داستان گویی در کودکان پرداخته و مناسبت استفاده از هر نوع ارزیابی را در بافتهای مختلف مورد بررسی قرار داده است. مواد و روش ها: جهت انجام مرور حاضر از كليد واژه هاي «مهارت های داستان گویی» ،«داستان گویی در کودکان»،«ارزیابی مهارت های زبانی»،«ارزیابی داستان گویی » براي جستجو در پايگاه هاي اطلاعاتي ، ASHA،Pubmed  ، Science direct ،Cochrane , Google scholar ،Wiley، Iran Medex ، Iran Doc ، SID  بین سالهای 1946- 2014 استفاده شد. 105 مقاله حاصل جستجوي فوق بود، كه بعد از مطالعه عنوان و خلاصه مقاله ها از 61 مورد از مرتبط ترين آن ها براي نگارش  این مقاله استفاده شده است. نتایج: روش های متنوعی برای تهیه ی نمونه ی گفتاری از داستان گویی کودک وجود دارد از جمله ، ساختن داستان، بازگویی داستان و  بازگویی تجربیات شخصی. در مقالاتی که مورد بررسی قرارگرفتند ابزارهای متفاوتی برای ارزیابی رسمی داستان گویی ذکرشده بودند که هرکدام از آنها جنبه های مختلفی از درک و بیان داستان گویی را بررسی می کنند همچنین این ابزارها از نظر بررسی ساختارهای  بزرگ و کوچک و نحوه ی نمره دهی نیز باهم متفاوتند شیوه ی نمره دهی همه ی این ابزارها به جز پروتکل ارزیابی داستان گویی (NAP) به روش نسخه نویسی انجام می شود. بحث: در برخی مطالعه ها نشان داده شده است که در دسترس بودن تصاویر و ارائه ی داستان، روایت پیچیده تر و بلندتری را ایجاد می کند. همچنین تکلیف ساختن داستان نسبت به تکلیف بازگویی سخت تر است و تفاوت ها را بیشتر آشکار می کند. درحال حاضر در ایران NAP ، SRT و ENNI دردسترس ترین روش هایی هستند که برای ارزیابی داستان گویی وجود دارد، مورداستفاده قرارگرفته است و می توان به آن ها استناد کرد. همچنین به نظر می رسد به دلیل اینکه ابزار NAP به نسخه نویسی نیاز ندارد، شیوه ی نمره دهی اش نسبت به دیگر ابزارها آسان تر باشد.
